# A new optimization algorithm based on average and subtraction of the best and worst members of the population for solving various optimization problems

**DOI:** 10.7717/peerj-cs.910

**Published:** 2022-03-07

**Authors:** Mohammad Dehghani, Štěpán Hubálovský, Pavel Trojovský

**Affiliations:** 1 Department of Mathematics/Faculty of Science, University of Hradec Králové, Hradec Kralove, Czech Republic; 2 Department of Applied Cybernetics/Faculty of Science, University of Hradec Králové, Hradec Kralove, Czech Republic

**Keywords:** Optimization, Optimization algorithm, Optimization problem, Algorithm of best and worst members of the population

## Abstract

In this paper, a novel evolutionary-based method, called Average and Subtraction-Based Optimizer (ASBO), is presented to attain suitable quasi-optimal solutions for various optimization problems. The core idea in the design of the ASBO is to use the average information and the subtraction of the best and worst population members for guiding the algorithm population in the problem search space. The proposed ASBO is mathematically modeled with the ability to solve optimization problems. Twenty-three test functions, including unimodal and multimodal functions, have been employed to evaluate ASBO’s performance in effectively solving optimization problems. The optimization results of the unimodal functions, which have only one main peak, show the high ASBO’s exploitation power in converging towards global optima. In addition, the optimization results of the high-dimensional multimodal functions and fixed-dimensional multimodal functions, which have several peaks and local optima, indicate the high exploration power of ASBO in accurately searching the problem-solving space and not getting stuck in nonoptimal peaks. The simulation results show the proper balance between exploration and exploitation in ASBO in order to discover and present the optimal solution. In addition, the results obtained from the implementation of ASBO in optimizing these objective functions are analyzed compared with the results of nine well-known metaheuristic algorithms. Analysis of the optimization results obtained from ASBO against the performance of the nine compared algorithms indicates the superiority and competitiveness of the proposed algorithm in providing more appropriate solutions.

## Introduction

### Motivation

Providing the best solution while respecting the limitations of the given problem is the main goal in optimization. An optimization problem can have several solutions that in order to compare these solutions and select the appropriate solution, the main criterion is the value of the objective function (Dhiman, 2021). Optimization is an important and critical activity in many fields of economics, industry and other sciences. The methods proposed to solve the optimization problems are deterministic methods and stochastic methods. In deterministic methods, gradient-based classes that use gradient information for finding the global optimal solution are the mathematical programming methods, containing nonlinear and linear programming (Faramarzi & Afshar, 2014), while nongradient-based classes use conditions for finding the global optimal solution (Lera & Sergeyev, 2018). One essential difficulty of mathematical programming approaches is the high probability of getting caught in local optimal solutions during the scan of the nonlinear search space. To overcome this potential pitfall, existing methods are modified or combined with other algorithms that are used only for certain problems.

Among the difficulties of nongradient-based deterministic methods are the hardness of implementation and the need a high level of mathematical knowledge in order to apply (Doumari et al., 2021b).

Many optimization problems are more complex than can be solved with classical and deterministic computational methods (Cavazzuti, 2013). One of the most promising and important study areas in recent years, has been the design of innovative stochastic methods called optimization algorithms. One of the available solutions to deal with such problems is the use of optimization algorithms. Another reason for using optimization algorithms is too much and impossible time of deterministic mathematical methods to solve optimization problems with many and complex parameters (Dhiman & Kumar, 2017). Optimization methods have similarities to social, natural and physical systems, as well as other processes that can be modeled as optimizers. The structure of these methods is derived from the optimization process in those systems, which have had good results in solving problems with complex structures (Dehghani, Hubálovský & Trojovský, 2021). In most of these methods, the search operation begins by producing a random population in the search area. Then, using the computational intelligence in the algorithm, the solutions are moved to the search space. This displacement is such that after passing through several iterations of the algorithm, the population converges toward the optimal point (Dehghani & Trojovský, 2021). The strategy of changing the status of population members and moving them in the search space is the most important difference of optimization algorithms. In recent years, the development and use of optimization algorithms has grown significantly.

### Research gaps

Global optimum is the best solution to an optimization problem. The most important challenge in optimization algorithms is that due to the randomness of the search process, the proposed solutions of these methods are not exactly the same as the global optimum. Therefore, the proposed solution to a given problem using optimization algorithm is a quasi-optimal which is at best equal to the global optimum (Dehghani et al., 2020c). Therefore, it can be said that a quasi-optimal solution that is closer to the global optimal is a more appropriate solution. This led researchers to develop numerous optimization algorithms to provide better quasi-optimal solutions.

Another important issue in optimization studies is the acceptance of the fact that there is no algorithm that has the best performance in solving all optimization problems. According to the no free lunch (NFL) theorem (Wolpert & Macready, 1997), if an algorithm is highly capable of solving one or more optimization problems, no assurance can be given that it can also solve other problems. NFL theorem encourages and requires scholars to design new algorithms for solving optimization problems in different applications.

### Contribution

A new evolutionary optimizer called Average and Subtraction-Based Optimizer (ASBO) is designed for use in solving various optimization problems in this paper. The scientific contribution of this research can be listed as follows:

- ASBO is designed based on the idea of using average information and subtracting the best and worst population members for guiding the population toward the optimal solution.
- The various steps of ASBO are expressed, and then the concepts expressed in these steps are mathematically modeled.
- Twenty-three standard benchmark functions including seven unimodal functions, six high dimensional multimodal functions, and 10 fixed dimensional multimodal functions have been employed for ASBO evaluation.
- The optimization results obtained from the implementation of ASBO in optimizing these objective functions are analyzed against the performance of nine well-known algorithms.
- The findings and simulation results indicate the capability of the proposed algorithm to effectively solve problems and its superiority over nine compared algorithms.

### Organization

The rest of the paper is organized as follows: In the second section a lecture review is studied; the problem definition and the formulation are presented in the third section; the proposed ASBO is introduced and modeled in the fourth section; ASBO simulation and optimization results are studied in the fifth section; the sixth section discusses the results; and finally, in the seventh section, conclusions and several perspectives for ASBO are provided.

## Literature Review

Stochastic optimization algorithms have shown an acceptable ability to effectively solve optimization problems by providing appropriate solutions. These algorithms can be employed in various issues such as cloud computing (Prakash & Bala, 2014a, 2014b; Prakash, Bawa & Garg, 2021), cross-platform applications (Vassallo et al., 2019), engineering (Dehghani et al., 2020b), energy commitment (Dehghani, Montazeri & Malik, 2019) and other optimization challenges in science fields. Stochastic optimization algorithms can be divided into four types, namely evolutionary, swarm, physics and game-based optimization algorithms with respect to the central idea in their design.

Evolutionary-based techniques are developed based on simulation of evolutionary theory and biology sciences. One of the most famous and oldest evolutionary algorithms that uses evolutionary biology techniques such as inheritance and mutation is the Genetic Algorithm (GA). GA is a programming method that applies genetic evolution and Darwin’s theory of evolution by natural selection as a problem-solving technique. Organisms that have more capabilities and abilities to perform activities in the environment will have a higher birth rate, and naturally, organisms that are less compatible with the environment will have a lower birth rate. After several periods of time and several generations, the population tends to have more organisms whose chromosomes are more compatible with the environment. Over time, the composition of the individuals in society changes due to natural selection, and this is a sign of population evolution (Goldberg & Holland, 1988). Evolution Strategy (ES) (Beyer & Schwefel, 2002), Genetic Programming (GP) (Banzhaf et al., 1998), Biogeography-based Optimizer (BBO) (Simon, 2008), and Differential Evolution (DE) (Storn & Price, 1997) are some other evolutionary-based algorithms.

Swarm-based techniques mimic various natural phenomena, swarming behavior of insects, animals, birds and other living things. Ant Colony Optimization (ACO) is introduced as a nature-based optimizer which based on imitations the behavior of ants. ACO uses simple agents called ants to find suitable solutions to optimization problems in a repetition-based process. Ants can find the shortest route from a food source to the nest using pheromone information. The ants pour pheromones on the ground while walking and follow the path by smelling the pheromone spilled on the ground. If they come to a crossroads on the way to the nest, they choose the path randomly since they have no information about a better way. On average, half of the ants are expected to choose the first path and the other half the second path. Because one path is shorter than the other, more ants pass through it, and more pheromones accumulate on it. After a short time, the number of pheromones on both paths reaches a level that influences the decision of new ants to choose a better path. From then on, newer ants are more likely to prefer the shorter path because they see more pheromones on the shorter path at the decision point. After a short time, all ants will choose this path (Dorigo, Maniezzo & Colorni, 1996). The idea behind the swarm movement of fish and birds led to the design of the famous Particle Swarm Optimization (PSO). Every population member in the PSO who is considered a particle is a candidate solution to the problem. These particles move in the search space according to the two main concepts of the experience of each particle as individual knowledge and the experience of the whole population as collective knowledge. As a result of this strategy, particles will tend to the optimal areas in the search space and will be able to provide an optimal solution to the given problem (Kennedy & Eberhart, 1995). The special potential that exists in the educational space of a classroom has led to the design of Teaching-Learning Based Optimization (TLBO). Simulation of teacher-learner interactions in the two phases of teaching and learning is the main inspiration of TLBO. In the training phase, the best member of the population is assigned as a teacher and the other members of the population are trained by the teacher as students in the class. In the second phase, called the learners phase, students try to improve each other’s situation by sharing information with each other (Rao, Savsani & Vakharia, 2011). Simulating animal behavior and strategy can be a motivator for metaheuristic design. In this regard, the behavior of gray wolves, whose leadership is determined by the introduction of four different types of wolves, has been used in the design of Gray Wolf Optimizer (GWO). The alpha type is the strongest wolf in the herd. Beta and delta are the second and third strongest wolves in the herd, respectively. The omega type also includes other wolves in the herd. The natural behavior of these wolves during hunting is modeled in three phases: prey search, prey siege, and finally prey attack (Mirjalili, Mirjalili & Lewis, 2014). Nutrition intake and hunting methods of humpback whales, known as bubble hunting are applied to design swarm-based Whale Optimization Algorithm (WOA). In this method of hunting, each whale releases air bubbles under the sea and creates walls of rising air in the water. The krill and fish that are inside the aerial wall go to the center of the bubble circle out of fear. The whale is then able to swallow a large number of them by opening its mouth. The humpback whales are able to detect the position of the prey and surround them. Because the optimal position in the search space is uncertain, in the WOA, it is assumed that the best current solution is the target prey, or it is a nearby point (Mirjalili & Lewis, 2016).

Imitation of the behavior of marine predators in the oceans that are able to find and trap prey was the impetus for the development of Marine Predators Algorithm (MPA). In general, most animals in the wild use the random walk strategy effectively to find food. Random walking is a random process in which the next situation depends on the current situation and the probability of moving to the next place, which is mathematically modeled. One of the most popular random walk classes is the Levy flight class, which is used in the design of the MPA to model the movement strategy of marine predators to trap prey (Faramarzi et al., 2020).

Another bioinspired optimization algorithm is Tunicate Swarm Algorithm (TSA) which have been proposed with inspiration of modelling the jet propulsion and the swarm actions of tunicates within the navigation and foraging procedure. An important characteristic of tunicates is their ability to find food sources at sea. This ability is similar to achieving the optimal solution in the search space of an optimization problem. When finding food sources, tunicate behavior is modeled based on three main conditions, namely, (i) avoiding conflicts between tunicates, (ii) moving toward the position of the best tunicate, and (iii) remaining close to the best tunicate, which have been used in the design of the TSA (Kaur et al., 2020). Some of other swarm-based algorithms include Spotted Hyena Optimizer (SHO) (Dhiman & Kumar, 2017), Artificial Ecosystem-based Optimization (AEO) (Zhao, Wang & Zhang, 2020), Cat- and Mouse-based Optimization (CMBO) (Dehghani, Hubálovský & Trojovský, 2021), Artificial Gorilla Troops Optimizer (AGTO) (Abdollahzadeh, Soleimanian Gharehchopogh & Mirjalili, 2021), Horse Herd Optimization Algorithm (HOA) (MiarNaeimi, Azizyan & Rashki, 2021), Aquila Optimizer (AO) (Abualigah et al., 2021), Golden Eagle Optimizer (GEO) (Mohammadi-Balani et al., 2021) and Mutated Leader Algorithm (MLA) (Zeidabadi et al., 2022).

Physics-based techniques have been developed with mathematical modeling of physical phenomena and laws. Gravitational Search Algorithm (GSA) is a physics-based optimizer that uses simulations of Newton’s law of gravitation and the laws of motion on a population of masses. In GSA, each mass portends a solution to the problem. These masses exert force on each other according to the law of gravity according to their distance from each other. Then, based on the modeling of the laws of motion, this population of masses moves toward the optimal areas in the search space (Rashedi, Nezamabadi-Pour & Saryazdi, 2009). Momentum Search Algorithm (MSA) is a physics-based algorithm that uses momentum law modeling and Newtonian laws of motion to design a stochastic optimizer. In the MSA, population members are bullets that are placed in the search space and move according to Newton’s laws of motion based on the momentum that animates them. Given that the momentum applied to the bullets is in the direction of the best solution, after a certain number of repetitions, the bullets converge toward the optimal solution (Dehghani & Samet, 2020). Spring Search Algorithm (SSA) is proposed based on the mathematical modeling of Hooke’s law in a system of springs and weights. In SSA, search agents are weights that apply elastic force to each other based on the springs to which they are attached. A weight that has a better status in the search space pulls other weights toward a better position by springs with more spring constants. In an iterative process, the weights are expected to converge toward the optimal solution (Dehghani et al., 2021; Dehghani et al., 2020e). Some of the other physics-based algorithms are: Flow Direction Algorithm (FDA) (Karami et al., 2021), Simulated Annealing (SA) (Fogel, Owens & Walsh, 1966), Electromagnetic Field Optimization (EFO) (Abedinpourshotorban et al., 2016), Lichtenberg Algorithm (LA) (Pereira et al., 2021), and Archimedes Optimization Algorithm (AOA) (Hashim et al., 2021).

Game-based techniques have been expanded based on simulating player behavior and rules in different games. Ring Toss Game-Based Optimization (RTGBO) is a game-inspired solver method which have been proposed respect to the ring throwing simulations and scoring rules in the ring toss game. In RTGBO, the search agents are the rings that are thrown toward the score bars in optimal areas. During the iterations of the algorithm, the rings converge toward the optimal solution (Doumari et al., 2021a). Hide Object Game Optimization (HOGO) models players’ behavior in finding an object hidden in the game space. From the algorithm’s point of view, the hidden object is the optimal solution that players, as the algorithm population, try to find. The transfer of information between players leads the algorithm to come closer to the optimal solution (Dehghani et al., 2020g). Darts Game Optimization (DGO) (Dehghani et al., 2020f), Tug of War Optimization (TWO) (Kaveh & Zolghadr, 2016), Football Game Based Optimizer (FGBO) (Dehghani et al., 2020a), and Volleyball Premier League (VPL) (Moghdani & Salimifard, 2018) are some of the other game-based algorithms.

## Problem Definition and Formulation

An optimization problem is one that has more than one feasible solution. A feasible solution is a solution that is calculated according to the constraints of the problem. The process of selecting the best solution among these feasible solutions is called optimization (Dehghani et al., 2020d). The criterion for selecting the best solution is the objective function value. Different optimization problems in terms of constraints are divided into the following two categories:

(A) Unconstrained optimization problems: The main goal in these problems is to minimize or maximize the objective function without any restrictions on the decision variables.
(B) Constrained optimization problems: In most practical problems, optimization is done according to some constraints. These constraints may exist in the behavior and performance of the system as well as in the physics and geometry of the problem.

Equations representing the constraints may be equality constraints or inequality constraints; in each case, the optimization method is different. However, the constraints determine the acceptable area in the design (Han et al., 2021).

An optimization problem is introduced from a general point of view using three sections: constraints, objective functions, and decision variables (Dhiman et al., 2020). An optimization problem can be modeled mathematically according to Eqs. (1)–(4).

(1) $${\rm Minimize/Maximize}:F(X)$$

Subject to:

(2) $${h_k}\left( X \right) = 0,k = 1,2, \ldots ,q,$$

(3) $${g_j}\left( X \right) \gt 0,j = 1,2, \ldots ,p,$$

(4) $$l{b_n} \le {x_n} \le u{b_n},n = 1,2, \ldots ,m,$$

where *F*(*X*) is the objective function, *h_k_* (*X*) is the *k*th inequality constraint, *q* is the number of equality constraints, *g_j_*(*X*) is the *j*th inequality constraint, *p* is the number of inequality constraints, *x_n_* is the *n*th problem variable, *lb_n_* is the lower bound, *ub_n_* is the upper bound of the *n*th problem variable and *m* is the number of problem variables.

The next step in the optimization process, after modeling, is to solve it effectively which can be calculated using optimization solving methods. Optimization algorithms are an effective and efficient stochastic technique which are able to present appropriate solutions to optimization problems. In the next section, ASBO method is introduced and designed.

## Average and Subtraction-Based Optimizer

This section introduces the proposed ASBO first and then mathematically models ASBO.

Each optimization problem has a palatable space for problem solutions called the search space. The search space can be visualized as a coordinate system with a number of axes equal to the problem decision variables. Population members move in this search space, aiming to reach the appropriate quasi-optimal solution. The values of the problem decision variables are determined by the position of the ASBO members in the search space. Each member of the population provides information to other members of the population about the situation in which they find themselves. In ASBO, in an iteration-based process, members of the population move to the optimal regions. The main idea in designing the proposed ASBO is to update the position of the population members of the algorithm based on the average information, and subtraction of the worst and best members of the population. After the full implementation of ASBO on the optimization problem, ASBO introduces the best solution obtained during the implementation process as the solution to the problem. The various ASBO steps are listed below:

Step 1: Specify the optimization problem and its information.
Step 2: Specify the parameters of the algorithm.
Step 3: Initial positioning of algorithm population members in the search space.
Step 4: Evaluate all members of the population.
Step 5: Determine the best and worst members of the population.
Step 6: Calculate the average and subtraction of the best and worst members of the population.
Step 7: Update ASBO’s population based on the average information and subtract the best and worst population members.
Step 8: Repeat steps 4 to 7 until the stop condition is reached.
Step 9: The best obtained quasi-optimal solution for the optimization problem is presented.

In ASBO, each population member is a feasible solution to the optimization problem. In fact, each ASBO member is mathematically a vector with the number of elements equal to the number of decision variables, while each element of this vector specifies the value of the variable corresponding to that element. The population members of ASBO are modeled according to Eq. (5).

(5) $${\rm{X  = }}{\left[ \matrix{
  {X_1} \hfill \cr 
   \vdots  \hfill \cr 
  {X_i} \hfill \cr 
   \vdots  \hfill \cr 
  {X_N} \hfill \cr}  \right]_{N \times m}} = {\left[ {\matrix{
   {{x_{1,1}}} &  \cdots  & {{x_{1,d}}} &  \cdots  & {{x_{1,m}}}  \cr 
    \vdots  &  \ddots  &  \vdots  &  {\mathinner{\mkern2mu\raise1pt\hbox{.}\mkern2mu \raise4pt\hbox{.}\mkern2mu\raise7pt\hbox{.}\mkern1mu}}  &  \vdots   \cr 
   {{x_{i,1}}} &  \cdots  & {{x_{i,d}}} &  \cdots  & {{x_{i,m}}}  \cr 
    \vdots  &  {\mathinner{\mkern2mu\raise1pt\hbox{.}\mkern2mu \raise4pt\hbox{.}\mkern2mu\raise7pt\hbox{.}\mkern1mu}}  &  \vdots  &  \ddots  &  \vdots   \cr 
   {{x_{N,1}}} &  \cdots  & {{x_{N,d}}} &  \cdots  & {{x_{N,m}}}  \cr 

 } } \right]_{N \times m}},$$

where, $X$ is the candidate solutions of ASBO, ${X_i}$ is the *i*th candidate solution of ASBO, $m$ is the number of decision variables of given problem, $N$ is the number of ASBO members, and ${x_{i,d}}$ is the value of the *d*th decision variables determined by the *i*th candidate solution.

Each ASBO searcher member is a potential solution to the given problem. By placing each of these solutions in the decision variables of the problem formula, the objective function is evaluated. This results in a value corresponding to each ASBO member for the objective function. The set of these values are modeled using a vector according to Eq. (6).

(6) $$F = {\left[ {\matrix{ {{F_1}} \cr \vdots \cr {{F_i}} \cr \vdots \cr {{F_N}} \cr } } \right]_{N \times 1}},$$

where ${F_i}$ represents the value of the objective function corresponding to the *i*th member, and $F$ denotes the set of these values together as the objective function vector. Comparing the values obtained for the objective function is the main criterion in determining the quality of solutions as well as identifying the worst and best ASBO members.

ASBO employs three different phases in the process of updating the algorithm population with the aim of improving candidate solutions.

In the first phase of ASBO, a member composed of the average of the best and worst members of the population is tasked with updating the ASBO population. This phase of ASBO is simulated based on Eqs. (7)–(9).

(7) $${L^{{P_1}}} = {{{X_b} + {X_w}} \over 2},$$

(8) $$x_{i,d}^{new,{P_1}} = \left\{ {\matrix{ {{x_{i,d}} + r \cdot \left( {L_d^{{P_1}} - I \cdot {x_{i,d}}} \right),}  &  {{F^{{P_1}}} \lt {F_i};} \cr {{x_{i,d}} + r \cdot \left( {{x_{i,d}} - L_d^{{P_1}}} \right),}  &  {else,} \cr } } \right.$$

(9) $${X_i} = \left\{ {\matrix{ {X_i^{new,{P_1}},}  &  {F_i^{new,{P_1}} \lt {F_i};} \cr {{X_i},}  &  {else,} \cr } } \right.$$

where $L^{P_1}$ is the average of the worst and best population members, $F^{P_1}$ is its objective function value, $L_d^{P_1}$ is the *d*th dimension of $L^{P_1}\!$, *X_b_* is the best member of ASBO, *X_w_* is the worst member of ASBO, $X_i^{new,P_1}$ is the new status of the *i*th population member based on phase 1, $F_i^{new,P_1}$ is its objective function value, $x_{i,d}^{new,P_1}$ is the *d*th dimension of $X_i^{new,P_1}$, *I* is a random number that is equal to 1 or 2, and *r* is a random number in interval [0, 1].

In the second phase, the position of the population members is updated based on the subtraction information of the best and worst population members. Concepts expressed in the second phase of ASBO is simulated using Eqs. (10)–(12).

(10) $${L^{{P_2}}} = {X_b} - {X_w},$$

(11) $$x_{i,d}^{new,{P_2}} = {x_{i,d}} + r \cdot L_d^{{P_2}},$$

(12) $${X_i} = \left\{ {\matrix{ {X_i^{new,{P_2}},}  &  {F_i^{new,{P_2}} \lt {F_i};} \cr {{X_i},}  &  {else,} \cr } } \right.$$

where $L^{P_2}$ is the subtraction of the worst and best members of ASBO, $X_i^{new,P_2}$ is the new proposed value of the *i*th candidate solution based on phase 2, $F_i^{new,P_2}$ is its objective function value and $x_{i,d}^{new,P_2}$ is the *d*th dimension of $X_i^{new,P_2}$.

Finally, in the third phase of ASBO, the best member is employed to lead the ASBO population to better solutions. This step of the update process in ASBO is simulated using Eqs. (13) and (14).

(13) $$x_{i,d}^{new,{P_3}} = {x_{i,d}} + r \cdot ({x_{i,d}} - I \cdot {x_{b,d}}),$$

(14) $${X_i} = \left\{ {\matrix{ {X_i^{new,{P_3}},}  &  {F_i^{new,{P_3}} \lt {F_i};} \cr {{X_i},}  &  {else,} \cr } } \right.$$

where $X_i^{new,P_3}$ is the new status of the *i*th population member based on phase 3, $F_i^{new,P_3}$ is its objective function value, and $x_{i,d}^{new,P_3}$ is the *d*th dimension of $X_{i}^{new,P_3}$.

**Scheme 1:**
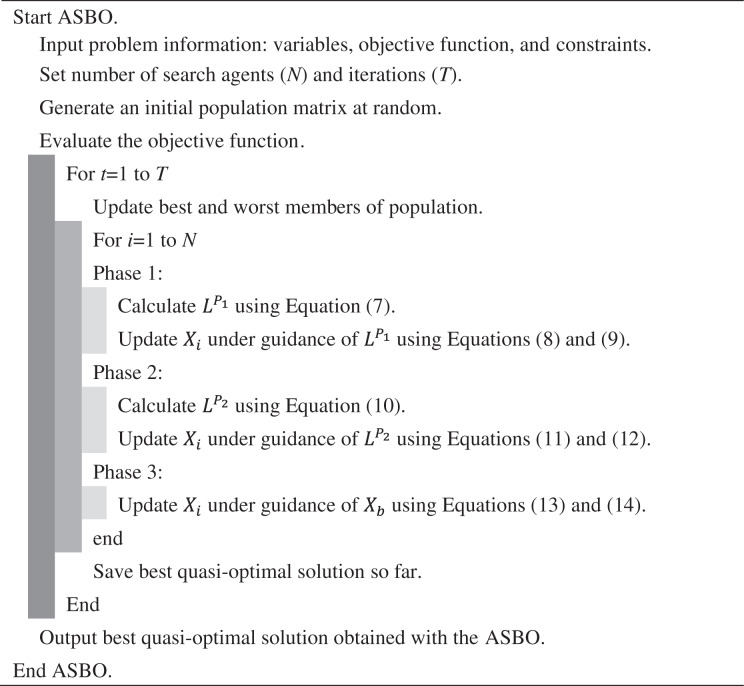
Pseudocode of ASBO. Pseudocode of algorithm based on average and subtraction of the best and worst members of the population.

**Figure 1:**
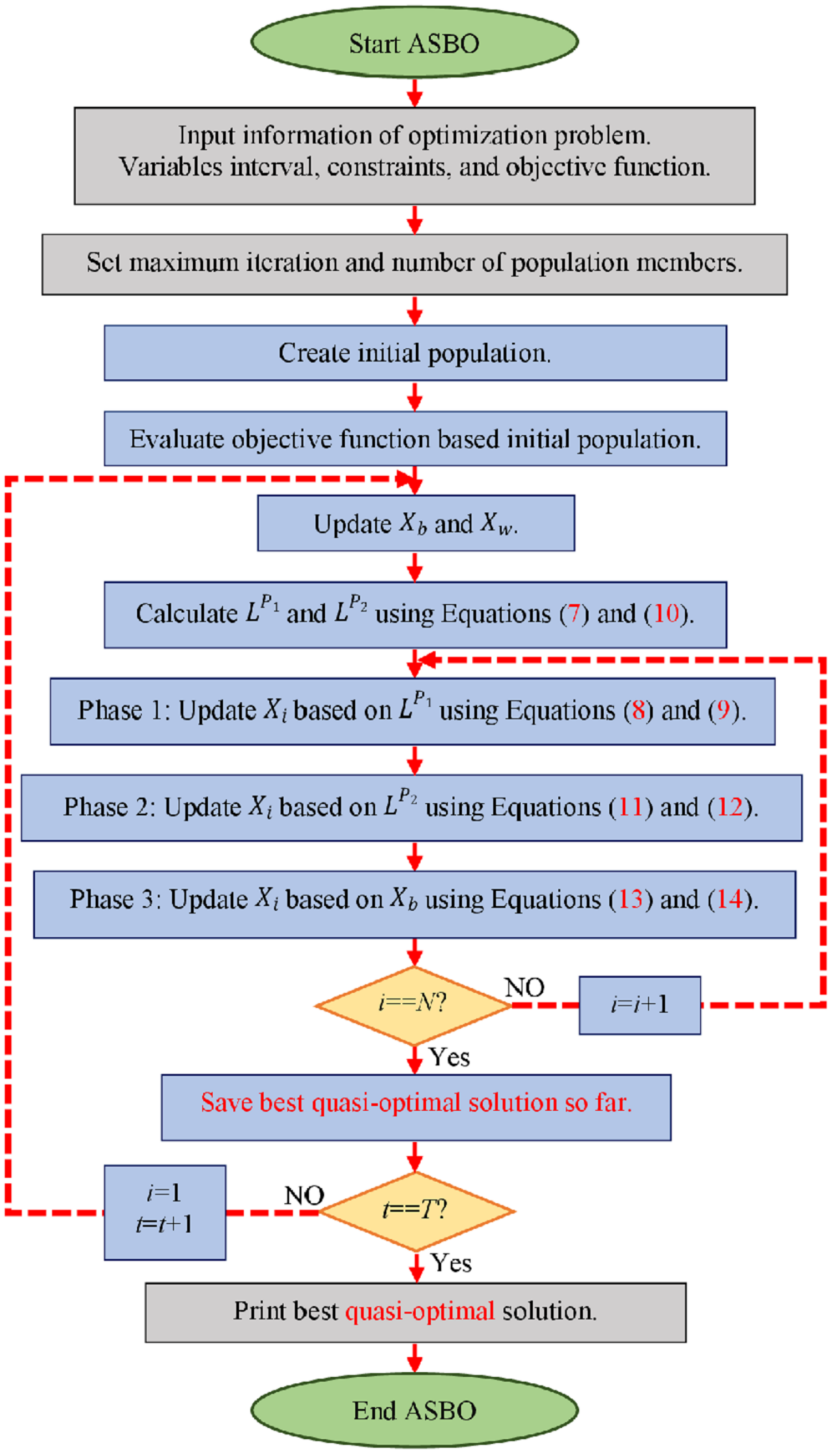
Flowchart of the proposed ASBO. Flowchart of the proposed algorithm based on average and subtraction of the best and worst members of the population for solving.

After implementing the described three phases of the proposed ASBO, each population member is placed in a new position in the search space. The new status of ASBO members means new candidate values for decision variables, leading to the evaluation of new values for the objective function. Based on the new values, the algorithm enters the next iteration, and the algorithm steps are repeated according to Eqs. (7)–(14) until the implementation of the algorithm is completed. After the complete implementation of ASBO, the best obtained solution during the iterations of the algorithm is introduced as the solution to the problem. The various steps of ASBO are presented as pseudocode in Scheme 1, and as flowcharts in Fig. 1.

In the proposed algorithm, the algorithm population is updated in three different phases. The first phase (which uses average information) and the second phase (which uses subtraction information) move the ASBO members in different areas of the search space and discover new areas. This update process increases the search power and exploration index in the proposed algorithm.

In the third phase, the position of the best member of ASBO is employed to guide the searcher members in the search space. After the algorithm identifies the optimal region based on its exploratory power, moving toward the best member causes the population members to converge toward the optimal solution. During the iteration of ASBO, as the amount of displacement of population members in the first and second phases decreases (because the worst and best members of the population approach each other), the algorithm moves to the best member of the population in smaller steps. This convergence process toward the optimal solution demonstrates the exploitation power of the proposed ASBO in achieving the appropriate solution to the optimization problem.

## Results

This section presents the ASBO experimental study on the effective solution of optimization problems and the quality analysis of optimization results *vs* the global optima. In this regard, seven unimodal test functions, six high dimensional multimodal functions, and 10 fixed dimensional multimodal functions have been employed for ASBO evaluation. Detailed information for these objective functions is specified in Tables 1 to 3 in Appendix A. Stochastic optimization algorithms will be able to succeed in optimization challenges when they have an acceptable power in the global search of problem-solving space to accurately scan different areas and identify the optimal area, as well as the appropriate power in the local search to converge to the global optimum. As a result, a successful optimization process occurs when the optimization algorithm has the right balance between global search and local search. The reason for choosing unimodal functions (including seven F1 to F7 test functions) is that these types of problems, with only one main peak in the search space, are very valuable choices in evaluating the local search of optimization algorithms. The main purpose of optimizing these problems is to analyze the ability of optimization algorithms in converging to the global optima. The choice of high-dimensional multimodal functions (including F8 to F13) is due to the fact that these types of functions, with multiple local optimal areas in the search space, challenge the ability of global search optimization algorithms. The main purpose of optimizing these types of problems is to evaluate the ability of optimization algorithms to cross non-optimal areas and thus identify the main optimal area. The reason for choosing fixed-dimensional multimodal functions (including F14 to F23) is that in this type of problem, it is important to identify the optimal region and domain of converging to the optimal solution simultaneously, which makes them suitable for analyzing the global search and local search capabilities of optimization algorithms. This type of problem evaluates the ability of optimization algorithms to strike the right balance between global search and local search. Additionally, to further analyze the quality of the proposed ASBO, the optimization results obtained are analyzed in comparison with the performance of nine algorithms namely SHO, PSO, TLBO, GA, WOA, TSA, GWO, GSA, and MPA. From the numerous optimization algorithms designed so far, nine methods have been selected for comparison with ASBO. The reason for choosing these nine competing algorithms is that GA and PSO are the best known and most widely used optimization algorithms. GSA, TLBO, and GWO, introduced between 2009 and 2014, have been popular methods for researchers and have been widely cited. WOA and SHO algorithms are among the most widely used techniques introduced in 2016 and 2017. MPA and TSA are recently developed optimizers that have quickly gained the attention of scientists and have been used in a variety of real-world applications. In presenting the optimization results, the criterion *ave* means the mean of the obtained solutions and the criterion *std* means the standard deviation of these solutions. In order to calculate these two criteria, Eqs. (15) and (16) have been used.

(15) $$ave = {1 \over {{N_r}}}\sum\limits_{i = 1}^{{N_r}} B Q{S_i},$$

(16) $$std = \sqrt {{1 \over {{N_r}}}\sum\limits_{i = 1}^{{N_r}} {{{\left( {BQ{S_i} - ave} \right)}^2}} },$$

where *N_r_* is the number of independent implementations and *BQS_i_* is the candidate solution in the *i*th execution.

Table 4 specifies the values set for the parameters of the compared algorithms.

**Table 4:** Parameter values for the comparative algorithms. Overvíew of parameter values for the used comparative algorithms.

Algorithm	Parameter	Value
GA		
	Type	Real coded
	Selection	Roulette wheel (Proportionate)
	Crossover	Whole arithmetic (Probability = 0.8, $\alpha = \left[ { - 0.5,1.5} \right]$)
	Mutation	Gaussian (Probability = 0.05)
PSO		
	Topology	Fully connected
	Cognitive and social constant	(*C*_1_, *C*_2_) = (2, 2)
	Inertia weight	Linear reduction from 0.9 to 0.1
	Velocity limit	10% of dimension range
GSA		
	Alpha, *G*_0_, *R*_*norm*_, *R*_*power*_	20, 100, 2, 1
TLBO		
	*T*_*F*_: teaching factor	*T*_*F*_ = round $\left[ {(1 + rand)} \right]$
	random number	*rand* is a random number between $0$ and $1$.
GWO		
	Convergence parameter (*a*)	*a*: Linear reduction from 2 to 0.
WOA		
	Convergence parameter (*a*)	*a*: Linear reduction from 2 to 0.
	*r* is a random vector in $\left[ {0,1} \right].$	
	*l* is a random number in $\left[ { - 1,1} \right].$	
TSA		
	P_min_ and P_max_	1, 4
	*c*1, *c*2, *c*3	random numbers lie in the range of $0 - 1.$
SHO		
	Control Parameter *(h)*	$\left[ {5,0} \right]$
	*M* constant	$\left[ {0.5,1} \right]$
MPA		
	Constant number	*P* = 0.5
	Random vector	*R* is a vector of uniform random numbers in $\left[ {0,1} \right].$
	Fish Aggregating Devices (*FADs*)	*FADs* = 0.2
	Binary vector	*U* = 0 or 1

The first group of functions that are selected for evaluation the efficiency of optimization algorithms in achieving suitable solutions are of the unimodal type. The optimization results of the F1 to F7 unimodal test functions using nine compared algorithms and the proposed ASBO are presented in Table 5.

**Table 5:** Optimization results. Optimization results of ASBO and other algorithms on unimodal function.

		ASBO	MPA	TSA	SHO	WOA	GWO	TLBO	GSA	PSO	GA
F_1_	ave	0	3.2715E−21	7.71E−38	3.19E−10	2.1741E−09	1.09E−58	8.3373E−60	2.0255E−17	1.7740E−05	13.2405
	std	0	4.6153E−21	7.00E−21	4.16E−19	7.3985E−25	5.1413E−74	4.9436E−76	1.1369E−32	6.4396E−21	4.7664E−15
F_2_	ave	1.59E−304	1.57E−12	8.48E−39	5.93E−09	0.5462	1.2952E−34	7.1704E−35	2.3702E−08	0.3411	2.4794
	std	0	1.42E−12	5.92E−41	9.82E−19	1.7377E−16	1.9127E−50	6.6936E−50	5.1789E−24	7.4476E−17	2.2342E−15
F_3_	ave	1.16E−264	0.0864	1.15E−21	4.80E−15	1.7634E−08	7.4091E−15	2.7531E−15	279.3439	589.4920	1536.8963
	std	0	0.1444	6.70E−21	4.96E−29	1.0357E−23	5.6446E−30	2.6459E−31	1.2075E−13	7.1179E−13	6.6095E−13
F_4_	ave	1.06E−252	2.6E−08	1.33E−23	1.02E−05	2.9009E−05	1.2599E−14	9.4199E−15	3.2547E−09	3.9634	2.0942
	std	0	9.25E−09	1.15E−22	1.32E−10	1.2121E−20	1.0583E−29	2.1167E−30	2.0346E−24	1.9860E−16	2.2342E−15
F_5_	ave	18.74776381	46.049	28.8615	28.8030	41.7767	26.8607	146.4564	36.10695	50.26245	310.4273
	std	2.38E−15	0.4219	4.76E−03	0.0150	2.5421E−14	0	1.9065E−14	3.0982E−14	1.5888E−14	2.0972E−13
F_6_	ave	0	0.398	7.10E−21	3.4142	1.6085E−09	0.6423	0.4435	0	20.25	14.55
	std	0	0.1914	1.12E−25	9.4278	4.6240E−25	6.2063E−17	4.2203E−16	0	7.5612E−04	3.1776E−15
F_7_	ave	2.00E−05	0.0018	3.72E−03	5.23E−05	0.0205	0.0008	0.0017	0.0206	0.1134	5.6799E−03
	std	3.64E−20	0.0010	5.09E−05	4.33E−09	1.5515E−18	7.2730E−20	3.87896E−19	2.7152E−18	4.3444E−17	7.7579E−19

Based on the results presented in this table, ASBO presents the global optima for the F1 and F6 functions. In addition, ASBO for F2 to F4 and F7 is the first best optimizer. Relying on the simulation results, it can be stated that ASBO has presented results that are superior and closer to the global optimum which has led to the dramatic superiority of ASBO over nine compared algorithms.

The second group of functions selected to evaluate the performance of optimization algorithms includes six high-dimensional multimodal functions. The ability of the optimization algorithms in solutions providing for F8 to F13 is presented in Table 6.

**Table 6:** Optimization results the 2nd group of functions. Optimization results of ASBO and other algorithms on high-dimensional function.

		ASBO	MPA	TSA	SHO	WOA	GWO	TLBO	GSA	PSO	GA
F_8_	ave	−6,000.5372	−3,594.16321	−5,740.3388	−2,677.4572	−1,663.9782	−5,885.1172	−7,408.6107	−2,849.0724	−6,908.6558	−8,184.4142
	std	4.68E−12	811.32651	41.5	2,97,834.0955	716.3492	467.5138	513.5784	264.3516	625.6248	833.2165
F_9_	ave	0	140.1238	5.70E−03	0	4.2011	8.5265E−15	10.2485	16.2675	57.0613	62.4114
	std	0	26.3124	1.46E−03	0	4.3692E−15	5.6446E−30	5.5608E−15	3.1776E−15	6.3552E−15	2.5421E−14
F_10_	ave	4.44E−15	9.6987E−12	9.80E−14	8.88E−15	0.3293	1.7053E−14	0.2757	3.5673E−09	2.1546	3.2218
	std	0	6.1325E−12	4.51E−12	2.05E−63	1.9860E−16	2.7517E−29	2.5641E−15	3.6992E−25	7.9441E−16	5.1636E−15
F_11_	ave	0	0	1.00E−07	0	0.1189	0.0037	0.6082	3.7375	0.0462	1.2302
	std	0	0	7.46E−07	0	8.9991E−17	1.2606E−18	1.9860E−16	2.7804E−15	3.1031E−18	8.4406E−16
F_12_	ave	1.15E−09	0.0851	0.0368	0.0194	1.7414	0.0372	0.0203	0.0362	0.4806	0.0470
	std	2.77E−24	0.0052	1.5461E−02	3.47E−06	8.1347E−12	4.3444E−17	7.7579E−16	6.2063E−17	1.8619E−16	4.6547E−17
F_13_	ave	1.41E−07	0.4901	2.9575	2.9532	0.3456	0.5763	0.3293	0.0020	0.5084	1.2085
	std	1.86E−17	0.1932	1.5682E−12	0.0006	3.25391E−12	2.4825E−15	2.1101E−14	4.2617E−14	4.9650E−15	3.2272E−14

The optimization results show that ASBO can present the global optimum for the F9 and F11. ASBO is the first-best optimizer for the F10, F12, and F13 functions. In the F8 optimization challenge, GA, TLBO, PSO, and ASBO are ranked first to fourth best optimizers, respectively. Analysis of the optimization results obtained for the F8 to F13 functions shows that ASBO has a higher ability than the nine compared algorithms.

The third group of functions that have been employed in this research to test the ability of optimization algorithms include ten fixed-dimensional multimodal functions. The implementation results of ASBO and the nine compared algorithms on these functions have been reported in Table 7.

**Table 7:** Optimization results the 3rd group. Optimization results of ASBO and other algorithms on fixed-dimensional function.

		ASBO	MPA	TSA	SHO	WOA	GWO	TLBO	GSA	PSO	GA
F_14_	ave	0.998	0.9980	1.9923	9.0060	0.9980	3.7408	2.2721	3.5913	2.1735	0.9986
	std	0	4.2735E−16	2.6548E−07	17.4188	9.4336E−16	6.4545E−15	1.9860E−16	7.9441E−16	7.9441E−16	1.5640E−15
F_15_	ave	0.0003	0.0030	0.0004	0.00031	0.0049	0.0063	0.0033	0.0024	0.0535	5.3952E−02
	std	0	4.0951E−15	9.0125E−04	1.43E−11	3.4910E−18	1.1636E−18	1.2218E−17	2.9092E−18	3.8789E−16	7.0791E−18
F_16_	ave	−1.03163	−1.0316	−1.0316	−0.9676	−1.0316	−1.0316	−1.0316	−1.0316	−1.0316	−1.0316
	std	1.95E−16	4.4652E−16	5.6514E−16	0.0100	9.9301E−16	3.9720E−16	1.4398E−15	5.9580E−16	3.4755E−16	7.9441E−16
F_17_	ave	0.3978	0.3979	0.3991	0.4432	0.4047	0.3978	0.3978	0.3978	0.7854	0.4369
	std	9.93E−17	9.1235E−15	2.1596E−16	0.0196	2.4825E−14	8.6888E−16	7.4476E−16	9.9301E−16	4.9650E−15	4.9650E−14
F_18_	ave	3	3	3	3	3	3.0000	3.0009	3	3	4.3592
	std	1.93E−16	1.9584E−15	2.6528E−15	3.7561E−15	5.6984E−15	2.0853E−15	1.5888E−15	6.9511E−16	3.6741E−15	5.9580E−16
F_19_	ave	−3.86278	−3.8627	−3.8066	−3.8005	−3.8627	−3.8621	−3.8609	−3.8627	−3.8627	−3.85434
	std	1.64E−15	4.2428E−15	2.6357E−15	0.0055	3.1916E−15	2.4825E−15	7.3483E−15	8.3413E−15	8.9371E−15	9.9301E−14
F_20_	ave	−3.322	−3.3211	−3.3206	−2.7924	−3.2424	−3.2523	−3.2014	−3.0396	−3.2619	−2.8239
	std	4.73E−16	1.1421E−11	5.6918E−15	0.0745	7.9441E−16	2.1846E−15	1.7874E−15	2.1846E−14	2.9790E−12	3.97205E−11
F_21_	ave	−10.1532	−10.1532	−5.5021	−4.1313	−7.4016	−9.6452	−9.1746	−5.1486	−5.3891	−4.3040
	std	1.59E−16	2.5361E−11	5.4615E−13	1.3660	2.3819E−11	6.5538E−15	8.5399E−15	2.9790E−14	1.4895E−13	1.5888E−12
F_22_	ave	−10.4029	−10.4029	−5.0625	−4.4760	−8.8165	−10.4025	−10.0389	−9.0239	−7.6323	−5.1174
	std	5.36E−16	2.8154E−11	8.4637E−14	2.5859	6.7524E−15	1.9860E−15	1.5292E−14	1.6484E−12	7.5888E−15	6.2909E−15
F_23_	ave	−10.5364	−10.5364	−10.3613	−4.3098	−10.0003	−10.1302	−9.2905	−8.9045	−6.1648	−6.5621
	std	6.16E−16	3.9861E−11	7.6492E−12	1.5908	9.1357E−15	4.5678E−15	6.1916E−15	7.1497E−14	2.7804E−15	3.8727E−15

What can be seen from the experimental results presented in this table is that ASBO has been able to discover the global optima in solving F14 and F15 with its best performance. ASBO has been able to rank the first best optimizer in solving F16, F19, and F20 in competition with nine compared algorithms. ASBO is also the number one optimizer in solving F17, F18, F21, F22, and F23 due to its smaller *std* index, regardless of the similarity in the *ave* index. Analysis of the optimization results of the F14 to F23 functions indicates that the proposed ASBO has a higher ability in providing suitable solutions against the compared algorithms.

**Figure 2:**
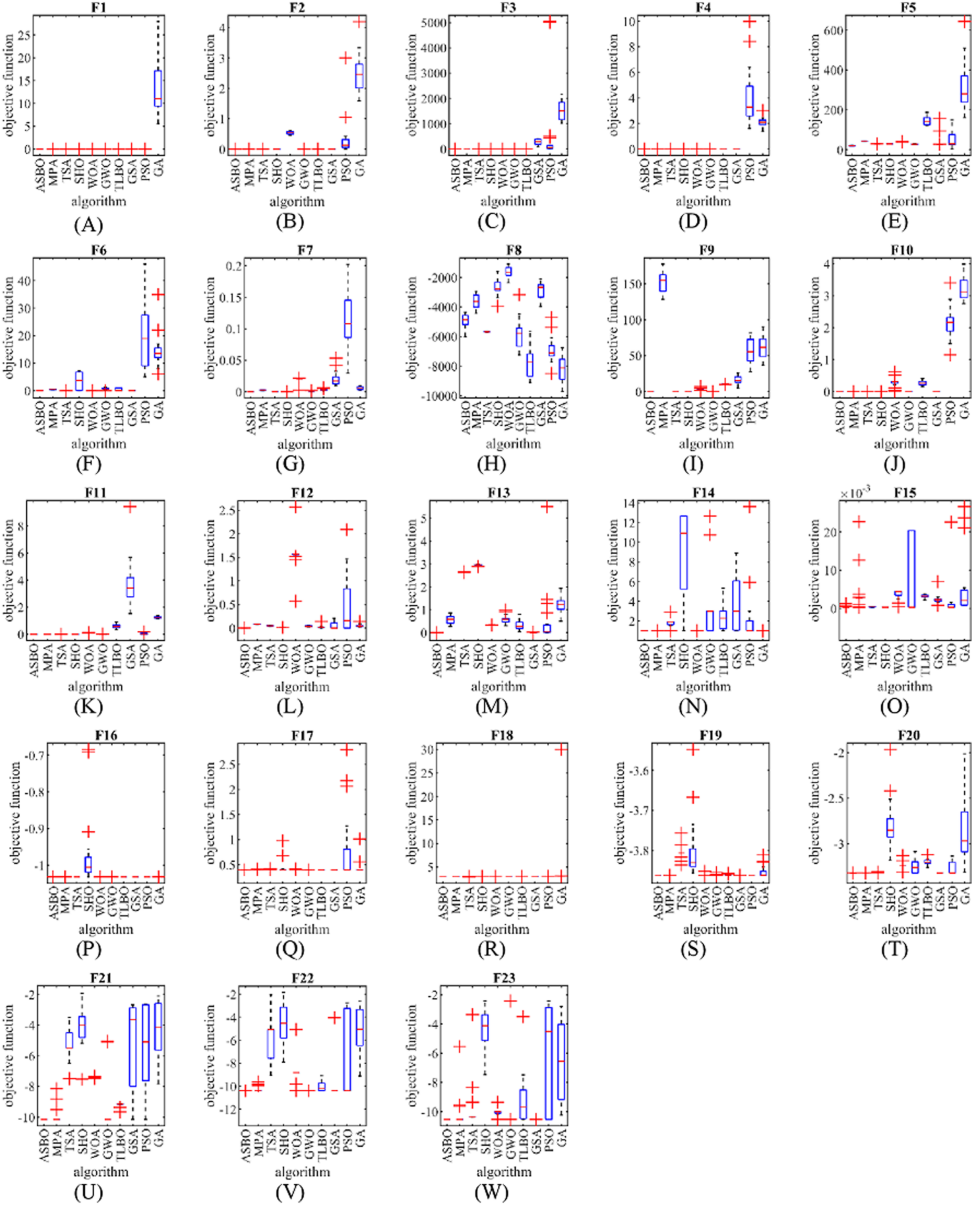
Boxplot of objective functions. Boxplot of composition objective function results for different optimization algorithms.

The performance of ASBO and the nine compared algorithms are presented as a boxplot in Fig. 2.

### Sensitivity analysis

The proposed ASBO algorithm completes its optimization operations by scan power of its searcher members in an iteration-based procedure. Therefore, any change in the number of ASBO population members or the number of ASBO iterations affects the output of this algorithm. This issue requires the study of ASBO sensitivity analysis with two parameters *N* and *T*. In this regard, a sensitivity analysis to evaluate the performance of ASBO under the influence of these two parameters is presented.

**Figure 3:**
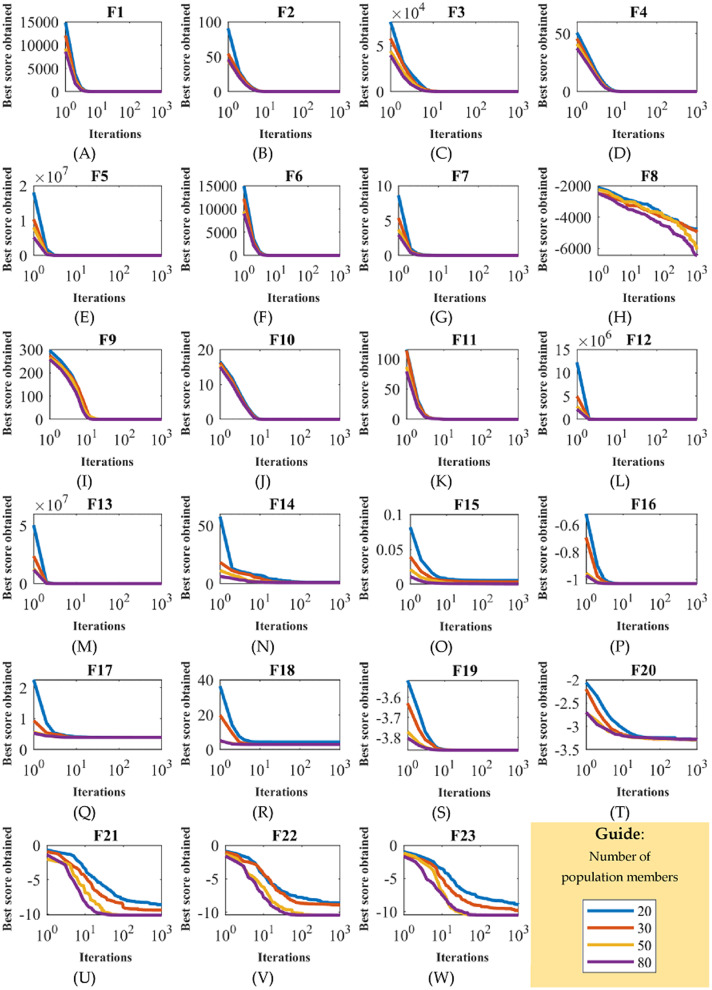
Sensitivity analysis of ASBO for number of population members. Sensitivity analysis of algorithm based on average and subtraction of the best and worst members of the population number of population members.

To analyze the sensitivity of ASBO to the N parameter, the algorithm is applied for four different values of N equal to 20, 30, 50 and 80 on solving the F1 to F23 test functions. Table 8 shows the results of ASBO sensitivity analysis to parameter N. The behavior of ASBO convergence curves under the influence of this analysis are shown in Fig. 3.

**Table 8:** Sensitivity analysis. Results of the algorithm sensitivity analysis to the number of population members.

Objective functions	Number of population members			
	20	30	50	80
F_1_	0	0	0	0
F_2_	6.3E−290	2.3E−299	1.5904E−304	8.8E−306
F_3_	3.3E−187	2.6E−206	1.1614E−264	2.4E−281
F_4_	2.5E−245	2.9E−251	1.0626E−252	1.3E−259
F_5_	24.23407	19.19636	18.74776381	18.31817
F_6_	0	0	0	0
F_7_	0.000211	0.000125	1.99654E−05	1.43E−05
F_8_	−5,176.3	−4,744.23	−6,000.537279	−6,586.72
F_9_	0	0	0	0
F_10_	4.44E−15	4.44E−15	4.44E−15	4.44E−15
F_11_	0	0	0	0
F_12_	0.005183	1.23E−12	1.15009E−09	1.06E−09
F_13_	0.195418	0.10718	1.4058E−07	7.02E−08
F_14_	1.14691	0.998	0.998	0.998
F_15_	0.005505	0.003366	0.0003	0.0003
F_16_	−1.03163	−1.03163	−1.03163	−1.03163
F_17_	0.397903	0.3978	0.3978	0.3978
F_18_	4.35	3	3	3
F_19_	−3.86278	−3.86278	−3.86278	−3.86278
F_20_	−3.2928	−3.29386	−3.322	−3.322
F_21_	−8.63809	−8.67736	−10.1532	−10.1532
F_22_	−8.57328	−8.87813	−10.4029	−10.4029
F_23_	−8.94325	−9.83606	−10.5364	−10.5364

Based on the simulation results, it is obtained that the increase in the number ASBO members has caused the search space to be scanned more accurately and the values of the objective function to be reduced by achieving more quasi-optimal solutions.

**Figure 4:**
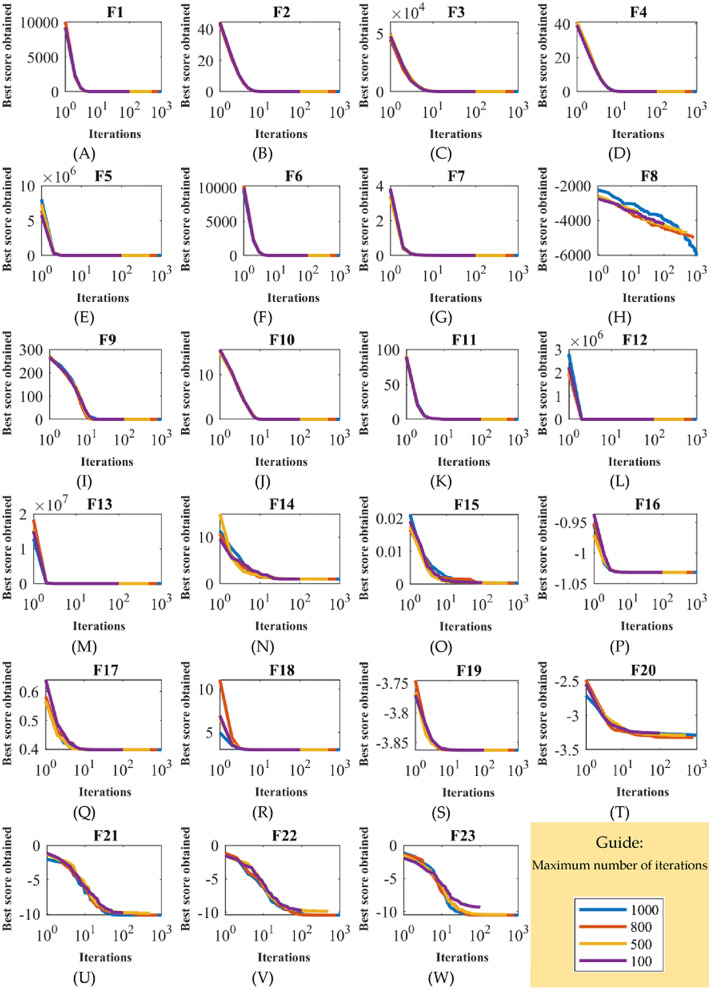
Sensitivity on number of iterations. Sensitivity analysis of algorithm based on average and subtraction of the best and worst members of the population for maximum number of iterations.

In the second study of sensitivity analysis, the performance of ASBO under the influence of changes in *T* parameter is investigated. In this experiment, ASBO is employed for different *T* values equal to 100, 500, 800, and 1,000 in solving F1 to F23. Table 9 presents the results of ASBO sensitivity analysis to parameter *T*. The behavior of ASBO convergence curves on the objective functions under the influence of the change in the maximum number of iterations of the algorithm is plotted in Fig. 4.

**Table 9:** Influence of the number of iterations. Results of the algorithm sensitivity analysis to the maximum number of iterations.

Objective functions	Maximum number of iterations			
	100	500	800	1,000
F_1_	5.94E−55	2.6E−289	0	0
F_2_	6.31E−29	1.6E−150	3.1E−241	1.5904E−304
F_3_	2.85E−20	5.9E−123	1.9E−201	1.1614E−264
F_4_	5.93E−24	1.9E−124	1.1E−199	1.0626E−252
F_5_	26.33555	22.72678	20.86457	18.74776381
F_6_	0	0	0	0
F_7_	0.000871	0.000188	8.84E−05	1.99654E−05
F_8_	−4,228.76	−4,708.57	−4,991.74	−6,000.537279
F_9_	0	0	0	0
F_10_	4.44E−15	4.44E−15	4.44E−15	4.44E−15
F_11_	5.9E−10	0	0	0
F_12_	0.020653	1.84E−05	1.39E−07	1.15009E−09
F_13_	0.556653	0.054633	0.04088	1.4058E−07
F_14_	0.998004	0.998004	0.998004	0.998
F_15_	0.000515	0.000374	0.001313	0.0003
F_16_	−1.03163	−1.03163	−1.03163	−1.03163
F_17_	0.39789	0.397887	0.397887	0.3978
F_18_	3	3	3	3
F_19_	−3.86278	−3.86278	−3.86278	−3.86278
F_20_	−3.25768	−3.29085	−3.27	−3.322
F_21_	−9.82098	−9.93551	−10.1520	−10.1532
F_22_	−9.73695	−9.84467	−10.4011	−10.4029
F_23_	−9.37413	−10.5358	−10.5361	−10.5364

What is clear from the simulation results is that increasing the *T* parameter improves the ASBO performance in converging to the global optima, thus reducing the values of the objective function.

### Statistical analysis

Validation of the efficiency of optimization algorithms based on the mean and standard deviation of their parameters provides valuable information for comparing their performance in optimization. Although unlikely, given that these algorithms are based in part on random population generation, it may happen that one algorithm will be better than the other algorithms only by chance. In this subsection, a statistical analysis of the performance of optimization algorithms is presented to determine whether the superiority of ASBO over competing algorithms is statistically significant. The Wilcoxon rank sum test (Wilcoxon, 1992), which is a non-parametric test, is employed for this purpose. In this analysis, an indicator called $p$-value determines whether the corresponding algorithm has a significant advantage over the alternative algorithm. The results of the Wilcoxon rank sum test with a confidence level of 0.95 are released in Table 10. What can be seen from the simulation results is that ASBO in all cases has a significant superiority over any of the competitor algorithms from a statistical point of view.

**Table 10:** Wilcoxon rank sum test results for ASBO against competitor algorithms.

Competitor algorithm	Test function type		
	Unimodal	High-dimensional multimodal	Fixed-dimensional multimodal
MPA	1.01E−24	4.02E−18	3.62E−10
TSA	1.48E−24	6.72E−05	4.68E−30
SHO	5.01E−14	1.24E−11	3.33E−33
WOA	9.78E−25	1.89E−21	4.75E−24
GWO	1.01E−24	4.34E−03	5.93E−30
TLBO	6.49E−23	2.07E−04	3.09E−26
GSA	1.97E−21	2.7E−12	3.32E−02
PSO	9.63E−22	7.59E−05	7.57E−06
GA	1.01E−24	1.93E−04	1.44E−34

## Discussion

Two valuable concepts that give optimization algorithms the ability to efficiently solve optimization problems are exploitation and exploration. It is by balancing these two concepts that optimization algorithms gain the ability to discover the optimal region and then converge to the optimal solution.

The concept of exploitation in the study of optimization algorithms expresses the ability of an algorithm to exact local search the problem-solving space of the optimization problem. Based on the exploitation concept, each optimization algorithm should be able to scan the neighborhood of the best solution obtained after identifying the optimal area in order to achieve better solutions with careful local search. Exploitation capability is an important feature for optimization algorithms, especially in solving optimization problems which have one main solution without any local optimal areas. The F1 to F7 unimodal test functions with this feature, are appropriate for assessing the exploitation ability of the optimization algorithms. Based on the optimization results of this type of function presented in Table 5, the proposed ASBO converges to solutions very close to the global optimum, and for the F1 and F6 functions even to the global optimum. This indicates the high power of ASBO in exploitation and local search. Analysis of the optimization results obtained from the nine compared algorithms against the results of ASBO indicate that the proposed algorithm has a much higher exploitation index than the compared algorithms.

The concept of exploration for an optimization algorithm is the ability to search different areas of the search space under the heading of global search. Global search allows the algorithm to get out of stopping in limited areas, especially local optimal areas. The exploration ability is an important feature of optimization algorithms, especially in solving problems which have local solutions in different areas of the search space. The F8 to F23 multimodal objective functions with this feature that also have local optimal solutions are proposed to assessment the exploration abilities of the optimization algorithms. According to the optimization results of the multimodal functions which are provided in Tables 3 and 4, it is determined that the proposed ASBO, with accurate scanning of the search space, is able to pass through the optimal local areas and move to the main solution of the objective functions; this holds, especially for the F9, F11, F14, and F15 functions, which have achieved the exact global optimum. Analysis of the performance results of the compared algorithms in optimizing multimodal functions indicates that the proposed ASBO has much higher capabilities in the exploration index and provides more appropriate solutions to optimization problems.

## Conclusions and Research Perspectives

In this paper, in order to effectively solve optimization problems, a new metaheuristic stochastic algorithm named Average and Subtraction Based Optimizer (ASBO) is designed. The fundamental idea in ASBO’s design is using the average information, and subtraction of the worst and best members of the population to guide the population to the optimal solution. The various steps of the proposed ASBO were explained, and mathematical model of proposed approach was presented for apply in solving optimization problems. The ASBO’s performance in presenting optimal solutions was tested on twenty-three standard unimodal and multimodal objective functions. The optimization results of the unimodal functions showed the high exploitation power of ASBO in converging toward global optima solution. The optimization results of the multimodal functions indicated the high exploration power of the proposed ASBO in accurate scanning of the search space and providing appropriate quasi-optimal solutions. Also, in order to analyze whether the results obtained from ASBO are significant or not, the proposed approach competed against the performance of nine algorithm, including SHO, PSO, TLBO, GA, WOA, TSA, GWO, GSA, and MPA. The simulation results showed that ASBO is an effective and efficient optimizer in solving optimization problems due to having a proper balance between exploration and exploitation. In addition, ASBO’s superior performance against nine compared algorithms indicated that ASBO was significantly competitive in providing solutions to optimization problems.

The authors would like to propose several research perspectives for future studies, including the design of binary version and multimodal version of the proposed ASBO. In addition, the application of ASBO in solving problems in various sciences and real-world problems are additional possibilities for further studies.

In future research plans, ASBO could be applied to optimize problems, and its results should be analyzed in comparison with other optimization algorithms. As a caveat for ASBO, and all optimization algorithms, there is always the possibility that newer optimization algorithms will be developed that will provide better quasi-optimal solutions.

## Abbreviations

The abbreviations used in this paper are listed in Table 11.

**Table 11:** Table with all abbreviations.

ASBO	Average and Subtraction-Based Optimizer
GA	Genetic Algorithm
PSO	Particle Swarm Optimization
TLBO	Teaching-Learning-Based Optimization
WOA	Whale Optimization Algorithm
GSA	Gravitational Search Algorithm
TSA	Tunicate Swarm Algorithm
GWO	Grey Wolf Optimizer
MPA	Marine Predators Algorithm
SHO	Spotted Hyena Optimizer
NFL	No Free Lunch
ES	Evolution Strategy
BBO	Biogeography-based Optimizer
DE	Differential Evolution
GP	Genetic Programming
ACO	Ant Colony Optimization
AEO	Artificial Ecosystem-based Optimization
CMBO	Cat and Mouse based Optimization
AGTO	Artificial Gorilla Troops Optimizer
HOA	Horse Herd Optimization Algorithm
AO	Aquila Optimizer
GEO	Golden Eagle Optimizer
MLA	Mutated Leader Algorithm
MSA	Momentum Search Algorithm
SSA	Spring Search Algorithm
FDA	Flow Direction Algorithm
SA	Simulated Annealing
EFO	Electromagnetic Field Optimization
LA	Lichtenberg Algorithm
AOA	Archimedes Optimization Algorithm
RTGBO	Ring Toss Game-Based Optimization
HOGO	Hide Object Game Optimization
DGO	Darts Game Optimization
VPL	Volleyball Premier League
FGBO	Football Game Based Optimizer
TWO	Tug of War Optimization

## Appendix A

Appendix A contains Tables 1–3 with the test functions we used in the paper.

**Table 1:** Unimodal test functions. Overview of unimodal test functions, which we used in our tests.

	Objective function	Range	Dimensions	F_min_
1.	${F_1}\left( x \right) = \sum\limits_{i = 1}^m {x_i^2}$	[−100, 100]	30	0
2.	${F_2}\left( x \right) = \sum\limits_{i = 1}^m {\left| {{x_i}} \right|} + \prod\limits_{i = 1}^m {\left| {{x_i}} \right|}$	[−10, 10]	30	0
3.	${F_3}\left( x \right) = \sum\limits_{i = 1}^m {{{\left( {\sum\limits_{j = 1}^i {{x_i}} } \right)}^2}}$	[−100, 100]	30	0
4.	${F_4}\left( x \right) = max\left\{ {\left| {{x_i}} \right|,1 \le i \le m} \right\}$	[−100, 100]	30	0
5.	${F_5}\left( x \right) = \sum\limits_{i = 1}^{m - 1}\left[100 \left({x_{i + 1}} - x_i^2 \right)^2\ +\ \left({{x_i} - 1} \right)^2\right]$	[−30, 30]	30	0
6.	${F_6}\left( x \right) = \sum\limits_{i = 1}^m {{{\left( {\left[ {{x_i} + 0.5} \right]} \right)}^2}}$	[−100, 100]	30	0
7.	${F_7}\left( x \right) = \sum\limits_{i = 1}^m i x_i^4 + random\left( {0,1} \right)$	[−1.28, 1.28]	30	0

**Table 2:** High dimensional multimodal test functions. Overview of High dimensional multimodal test functions, which we used in our tests.

	Objective function	Range	Dimensions	F_min_
8.	${F_8}\left( x \right) = \sum\limits_{i = 1}^m - {x_i}\sin \left( {\sqrt {|{x_i}|} } \right)$	$\left[ { - 500,500} \right]$	30	−1.2569e+04
9.	${F_9}\left( x \right) = \sum\limits_{i = 1}^m {\left[ {x_i^2 - 10\cos \left( {2\pi {x_i}} \right) + 10} \right]}$	$\left[ { - 5.12,5.12} \right]$	30	0
10.	${F_{10}}\left( x \right) = - 20\exp \left( { - 0.2\sqrt {{1 \over m}\sum\limits_{i = 1}^m {x_i^2} } } \right) - \exp \left( {{1 \over m}\sum\limits_{i = 1}^m {\cos } \left( {2\pi {x_i}} \right)} \right) + 20 + e$	$\left[ { - 32,32} \right]$	30	0
11.	${F_{11}}\left( x \right) = {\displaystyle{1 \over {4000}}}\sum\limits_{i = 1}^m {x_i^2} - \prod\limits_{i = 1}^m c os\left( {{{{x_i}} \over {\sqrt i }}} \right) + 1$	$\left[ { - 600,600} \right]$	30	0
12.	$\matrix{ {{F_{12}}\left( x \right) = {\displaystyle \pi \over m}\left\{ {10\sin \left( {\pi {y_1}} \right) + \sum\limits_{i = 1}^m {{{\left( {{y_i} - 1} \right)}^2}} \left[ {1 + 10{{\sin }^2}\left( {\pi {y_{i + 1}}} \right)} \right] + {{\left( {{y_n} - 1} \right)}^2}} \right\} + \sum\limits_{i = 1}^m u \left( {{x_i},10,100,4} \right)} \hfill \cr }$ $$u({x_i,a,i,n}) = \left\{ {\matrix{ {k(x_i-a)^n}, & x_i \gt -a; \cr 0, & -a\lt x_i \lt a; \cr {k(-x_i-a)^n}, & {x_i \lt-a}. \cr } } \right.	$\left[ { - 50,50} \right]$	30	0
13.	$\eqalign{ {F_{13}}\left( x \right) = 0.1\left\{ {{{\sin }^2}\left( {3\pi {x_1}} \right) + \sum\limits_{i = 1}^m {{{\left( {{x_i} - 1} \right)}^2}} \left[ {1 + {{\sin }^2}\left( {3\pi {x_i} + 1} \right)} \right] + {{\left( {{x_n} - 1} \right)}^2}\left[ {1 + {{\sin }^2}\left( {2\pi {x_m}} \right)} \right]} \right\} \cr\quad+ \sum\limits_{i = 1}^m u \left( {{x_i},5,100,4} \right)}$	$\left[ { - 50,50} \right]$	30	0

**Table 3:** Fixed dimensional multimodal test functions. Overview of Fixed dimensional multimodal test functions, which we used in out tests.

	Objective function	Range	Dimensions	F_min_
14.	${F_{14}}\left( x \right) = {\left( {{1 \over {500}} + \sum\limits_{j = 1}^{25} {{1 \over {j + \sum\limits_{i = 1}^2 {{{\left( {{x_i} - {a_{ij}}} \right)}^6}} }}} } \right)^{ - 1}}$	$\left[ { - 65.53,65.53} \right]$	2	0.998
15.	${F_{15}}\left( x \right) = \sum\limits_{i = 1}^{11} {{{\left[ {{a_i} - {{{x_1}\left( {b_i^2\ +\ {b_i}{x_2}} \right)} \over {b_i^2\ +\ {b_i}{x_3}\ +\ {x_4}}}} \right]}^2}}$	$\left[ { - 5,5} \right]$	4	0.00030
16.	${F_{16}}\left( x \right) = 4x_1^2 - 2.1x_1^4 + {1 \over 3}x_1^6 + {x_1}{x_2} - 4x_2^2 + 4x_2^4$	$\left[ { - 5,5} \right]$	2	−1.0316
17.	${F_{17}}\left( x \right) = {\left( {{x_2} - {{5.1} \over {4{\pi ^2}}}x_1^2 + {5 \over \pi }{x_1} - 6} \right)^2} + 10\left( {1 - {1 \over {8\pi }}} \right)cos{x_1} + 10$	[−5, 10] × [0, 15]	2	0.398
18.	$\eqalign{ {F_{18}}\left( x \right) = \left[ {1 + {{\left( {{x_1} + {x_2} + 1} \right)}^2}\left( {19 - 14{x_1} + 3x_1^2 - 14{x_2} + 6{x_1}{x_2} + 3x_2^2} \right)} \right] \cr\quad \times \left[ {30 + {{\left( {2{x_1} - 3{x_2}} \right)}^2} \times \left( {18 - 32{x_1} + 12x_1^2 + 48{x_2} - 36{x_1}{x_2} + 27x_2^2} \right)} \right]}$	$\left[ { - 5,5} \right]$	2	3
19.	${F_{19}}\left( x \right) = - \sum\limits_{i = 1}^4 {{c_i}} \exp \left( { - \sum\limits_{j = 1}^3 {{a_{ij}}} {{\left( {{x_j} - {P_{ij}}} \right)}^2}} \right)$	$\left[ {0,1} \right]$	3	−3.86
20.	${F_{20}}\left( x \right) = - \sum\limits_{i = 1}^4 {{c_i}} \exp \left( { - \sum\limits_{j = 1}^6 {{a_{ij}}} {{\left( {{x_j} - {P_{ij}}} \right)}^2}} \right)$	$\left[ {0,1} \right]$	6	−3.22
21.	${F_{21}}\left( x \right) = - \sum\limits_{i = 1}^5 {{{\left[ {\left( {X - {a_i}} \right){{\left( {X - {a_i}} \right)}^T} + 6{c_i}} \right]}^{ - 1}}}$	$\left[ {0,10} \right]$	4	−10.1532
22.	${F_{22}}\left( x \right) = - \sum\limits_{i = 1}^7 {{{\left[ {\left( {X - {a_i}} \right){{\left( {X - {a_i}} \right)}^T} + 6{c_i}} \right]}^{ - 1}}}$	$\left[ {0,10} \right]$	4	−10.4029
23.	${F_{23}}\left( x \right) = - \sum\limits_{i = 1}^{10} {{{\left[ {\left( {X - {a_i}} \right){{\left( {X - {a_i}} \right)}^T} + 6{c_i}} \right]}^{ - 1}}}$	$\left[ {0,10} \right]$	4	−10.5364

## Supplemental Information

10.7717/peerj-cs.910/supp-1 Supplemental Information 1 Main file with code of the proposed ASBO algorithm.

10.7717/peerj-cs.910/supp-2 Supplemental Information 2 The MATLAB codes of Executing the main file1. Includes the number of decision variables and the allowable range of variables for each objective function.

10.7717/peerj-cs.910/supp-3 Supplemental Information 3 The MATLAB codes of Executing the main file 2. The objective functions used for optimization.

## Additional Information and Declarations

### Data Availability

The following information was supplied regarding data availability: The MATLAB codes are available in the Supplemental Files.
